# Bacteriologic Profile and Antibiogram of Blood Culture Isolates in a Pediatric Care Unit

**DOI:** 10.4103/0974-2727.72156

**Published:** 2010

**Authors:** Kavitha Prabhu, Sevitha Bhat, Sunil Rao

**Affiliations:** Department of Microbiology, Yenepoya Medical College, Deralakatte, Mangalore, India

**Keywords:** Sepsis, methicillin resistant *Staphylococcus aureus*, extended spectrum beta lactamases, blood culture

## Abstract

**Background / Aims:**

Septicemia is one of the important causes of mortality and morbidity in neonates and children. Blood culture is the gold standard for the diagnosis. Emergence of multidrug resistant bacterial strains is a major problem in the management of sepsis. Present study was undertaken to identify the common bacterial pathogens associated with pediatric sepsis and to determine their antibiotic susceptibility pattern.

**Materials and Methods:**

Blood cultures from 185 suspected cases of sepsis were examined. The growths from the subcultures were identified by conventional biochemical tests. Antibiotic susceptibility testing was performed by modified Kirby-Bauer disk diffusion method and drug resistant strains in primary screening were further processed for extended spectrum beta lactamases (ESBL) and methicillin resistant *Staphylococcus aureus* (MRSA) status by combination disk method (ESBL) and oxacillin disk diffusion method (MRSA).

**Results:**

Out of the 185 cultures obtained from suspected cases, 81 (44%) were culture positive. Fifty-two (35%) of the culture isolates were Gram negative bacilli. Twenty-eight (64%) of the isolates were Gram positive cocci. One case was of mixed infection. The prevalence of MRSA in 41 strains of *S. aureus* was found to be 29% (12 strains). The overall prevalence of ESBL producers among 28 Gram negative bacterial isolates was found to be 32% (9 strains).

**Conclusion:**

This study stresses the need for the continuous screening and surveillance for antibiotic resistance in pediatric care unit.

## INTRODUCTION

Blood stream infections are very common in the pediatric age group and these are one of the common causes of morbidity and mortality in neonates and children. The rate of blood stream infections in children is about 20–50% in developing countries.[1,2]

Several risk factors have been identified both in the neonates and children, which make them susceptible to infections. The risk factors for neonatal septicemia include premature rupture of membrane, prolonged rupture, prematurity, Urinary Tract Infection, poor maternal nutrition, low birth weight, birth asphyxia and congenital anomalies.[1] The children at risk of septicemia include infants, children with serious injury and children on chronic antibacterial therapy, malnourished children, children with chronic medical problems, and children with immunosuppressants. Polymicrobial sepsis occurs in high risk patients and is associated with catheters, gastrointestinal diseases, neutropenia and malignancy.[3]

A wide variety of bacteria are involved in blood stream infections, the majority belonging to groups of bacteria, such as streptococci/enterococci, staphylococci or enterobacteriaceae.[4]

Children with septicemia present with fever, difficulty in breathing, tachycardia, malaise, refusal of feeds or lethargy.[5] They can have serious consequences like shock, multiple organ failure, disseminated intravascular coagulation, etc. Thus, the blood stream infections constitute one of the most serious situations and, as a result, timely detection and identification of blood stream pathogen is important.[6] Clinical assessment using a combination of symptoms and signs is a useful guide for the provisional diagnosis of septicemia. But bacteriologic culture to isolate the offending pathogen remains the mainstay of definitive diagnosis of septicemia.[1]

Knowledge of epidemiological and antimicrobial susceptibility pattern of common pathogens in a given area helps to inform the choice of antibiotics. We report the pattern of bacterial isolates in children with clinical diagnosis of septicemia, seen in the pediatric unit at Yenepoya University Teaching Hospital in Mangalore.

## MATERIALS AND METHODS

The present study was carried out between August 2009 and March 2010 in the Department of Microbiology, Yenepoya Medical College, Mangalore.

Blood for culture was collected from 185 clinically diagnosed septicemia cases following strict aseptic precautions. One milliliter (neonates) and 5 ml (children) blood was collected and inoculated into 10 and 50 ml, respectively, of brain heart infusion broth (1:10 dilution). The culture bottles were incubated at 37°C aerobically and periodic subcultures were done onto Mac Conkey’s agar, blood agar and chocolate agar after overnight incubation on day 3, day 4 and finally on day 7. The growth obtained was identified by conventional biochemical tests.

### Antibiotic susceptibility test

The standard disk diffusion test for susceptibility to routine antibiotics was done by modified Kirby-Bauer method. Zone sizes were measured and interpreted according to CLSI standards.[7] Drug resistant strains in primary screening were further processed for the detection of extended spectrum beta lactamases (ESBL) in Gram negative bacterial isolates and methicillin resistance in *Staphylococcus aureus* (MRSA) strains.

Detection of MRSA was done by oxacillin disk diffusion method by placing 1 μg oxacillin disk on the bacterial lawn culture of *S.aureus*. After overnight incubation, the zone of inhibition was measured. An inhibition zone of diameter less than or equal to 10 mm indicates MRSA. *S.aureus* ATCC 25923 was used as quality control for oxacillin susceptibility.[8]

ESBL producers were detected by combination disk method using cefotaxime (30 μg) and cefotaxime/clavulanate (30/10 μg) (HimediaMumbi, India). An increase of 5 mm in the zone of inhibition in a disk containing clavulanate compared to the drug alone was considered as positive for ESBL producers.[9]

## RESULTS

During the 6 month study period, 185 blood cultures were analyzed. Among them, 86 were from neonates. Eighty-one samples showed growth and 104 samples were negative.

Most infections were due to a single organism, while one case was a mixed infection by *Klebsiella pneumoniae* and *Enterococcus faecalis*. This was from a hospitalized patient.

Gram positive bacteria were encountered more often (64.19%) than Gram negative organisms (34.56%). The distribution of species of 185 isolates is reported in Table 1. Antibacterial resistance pattern of the Gram positive and Gram negative blood stream isolates are shown in Tables 2 and 3, respectively.

**Table 1:** Incidence and distribution of microorganisms isolated from blood culture

Bacterial isolates	Number (%)
*S. aureus*	41(50.61)
Coagulase negative staphylococci	10 (12.3)
*K. pneumoniae*	10 (12.3)
*Salmonella typhi*	7(8.64)
*Pseudomonas aeruginosa*	5(6.17)
*Acinetobacter* sp.	3 (3.70)
*Escherichia coli*	1 (1.23)
*Enterobacter species*	1 (1.23)
*Proteus mirabilis*	1 (1.23)
*Viridans streptococci*	1 (1.23)
Mixed (*Klebsiella* species and *enterococcus* species)	1 (1.23)
Total	81

**Table 2:** Antibacterial resistance pattern of the Gram positive blood stream isolates

	P/A	Ox	Cf	Va	G	Ce	Ak	Co
*S. aureus* (*n* =41)	26 (63.4)	12 (29.26)	5(12.19)	0 (0)	2 (4.87)	5 (12.19)	1 (2.43)	5 (12.19)
Coagulase negative staphylococci (*n* =10)	4 (40)	3 (30)	1 (10)	2 (20)	5 (50)	2 (20)	0 (0)	NT
*E.faecalis* (*n* =1)	1 (100)	NT	0 (0)	0 (0)	0 (0) (high level aminoglycoside)	0 (0)	NT	NT

P/A = penicillin/ampicillin, Ox = oxacillin, Cf = ciprofloxacin, Va = vancomycin, G = gentamycin, Ce = cefotaxime, Ak = amikacin, Co = cotrimoxazole, NT = not tested; Figures in parenthesis are in percentage

**Table 3:** Antibacterial resistance pattern of the Gram negative blood stream isolates

Antibiotic	Enterobacteriaceae except *S. typhi* (*n*=13)	*S. typhi* (*n*=7)	Non-fermenters (*n*=8
A	9 (69.2)	3 (42.85)	6 (75)
Ac	6 (46.15)	1 (14.28)	NT
Cn	5 (38.46)	NT	NT
Cu	NT	1 (14.28)	NT
Ce	6 (46.15)	0	3 (37.5)
Ca	7 (53.84)	NT	5 (62.5)
G	1 (7.69)	0	0
Ak	0	0	0
Cf	1 (7.69)	1 (14.28)	0
C	NT	0	0
Co	NT	0	4 (50)
Pt	1 (7.69)	NT	1 (12.5)
I	0	NT	0

A = ampicillin, Ac = amoxyclav, Cn = cephalothin, Cu = cefuroxime, Ce = cefotaxime, Ca = ceftazidime, G = gentamycin, Ak = amikacin, Cf = ciprofloxacin, C = chloramphenicol, Co = cotrimoxazole, Pt = piperacillin tazobactam, I = imipenem, NT = not tested; Figures in parenthesis are in percentage

## DISCUSSION

Severe sepsis remains one of the leading causes of death in children. Physical signs and symptoms, though useful in identifying possible cases, have limited specificity. Definitive diagnosis is by bacteriologic culture of blood samples to identify organisms and establish antibiotic susceptibility.

We processed 185 blood samples from clinically diagnosed septicemia cases. The rate of bacterial isolation in blood culture in this study was 43.78%, and is comparable to that of previous studies.[1] The weaker immune system in neonates and children explains this higher rate of isolation.

The common isolates in blood culture in our study were *S. aureus* 41 (50.61%), coagulase negative staphylococci 10 (12.3%) and *K. pneumoniae* 10 (12.3%). The findings are consistent with those of the previous studies.[10] This suggests that infections by these agents constitute a significant threat to child survival in this locale and other developing country settings.

In the management of sepsis in pediatric age group, empirical antibiotic therapy should be unit-specific and determined by the prevalent spectrum of etiological agents and their antibiotic sensitivity pattern.

Among Gram negative organisms, high resistance was noted against ampicillin (64.28%). Chloramphenicol and cefotaxime were very effective against *S.typhi* (100%). In other members of the family Enterobacteriaceae except. *S.typhi*, high resistance was seen to third generation cephalosporins (53.8%). For multiple resistant strains of Gram negative bacilli, a combination of a third generation cephalosporin (cefotaxime or ceftazidime) with amikacin may be appropriate.

However, recent reports suggest that at least 60–70% of the Gram negative organisms are resistant to the above antibiotics and routine use of these antibiotics might increase the risk of infections with ESBL positive organisms.[11]

In our study, out of the 28 strains of Gram negative isolates tested for ESBL production, 9 strains (32.14%) were ESBL producers. Thus, there is a need for continuous screening and surveillance for ESBL producers in pediatric unit.

Imipenem and piperacillin tazobactam proved to be the most effective antibiotics for all the Gram negative bacterial isolates, including non-fermenters, in units with higher incidence of resistant strains.

Also, 63.4% of the *S.aureus* strains showed resistance to penicillin. Penicillin resistant *S.aureus* bacteria are treated with cloxacillin, nafcillin or methicillin, but the worrisome fact is the emergence of MRSA.

In this study, the incidence of MRSA in 28 *S. aureus* strains was 29.26% (12 strains).The occurrence of MRSA is more common because of indiscriminate use of higher antibiotics as an emergency empirical therapy. Vancomycin remains the drug of choice for the Gram positive bacterial isolates in our setup. MRSA should be treated with a combination of ciprofloxacin or vancomycin with amikacin.

This study has shown that *S. aureus* and Gram negative rods including *K. pneumoniae* are the leading causes of septicemia in pediatric age group, a pattern similar to that of other low income countries. Observed decline in susceptibility of these common pathogens to common antibiotics calls for increased efforts to ensure more rational use of these drugs.

The main forces driving the increase in antimicrobial resistant bacteria are poor infection control practices and inappropriate use of antibiotics. Specific antibiotic utilization strategies like antibiotic restriction, combination therapy and antibiotic cycling may help to decrease or prevent the emergence of resistance.
